# Effect of a Multi-Species Direct-Fed Microbial on Growth Performance, Nutrient Digestibility, Intestinal Morphology and Colonic Volatile Fatty Acids in Weanling Pigs

**DOI:** 10.3390/ani14121749

**Published:** 2024-06-10

**Authors:** Panumas Kongpanna, John A. Doerr, Dachrit Nilubol, Uttra Jamikorn

**Affiliations:** 1 Department of Animal Husbandry, Faculty of Veterinary Science, Chulalongkorn University, Bangkok 10330, Thailand; panumaskongpanna@gmail.com; 2 Agrarian Solutions, 585 Shawnee St., Nappanee, IN 46550, USA; jadoerr2@gmail.com; 3 Department of Veterinary Microbiology, Faculty of Veterinary Science, Chulalongkorn University, Bangkok 10330, Thailand

**Keywords:** apparent ileal digestibility, growth performance, direct fed microbial, volatile fatty acids, weanling pigs

## Abstract

**Simple Summary:**

Supplementing the ration of weanling pigs for 6 weeks with a novel commercial multi-species direct-fed microbial (DFM) based on cell-wall deficient *Lactobacillus* spp. and *Bacillus subtilis* showed a greater apparent ileal digestibility (AID) of essential amino acids (EAAs) consisting of Arg, Ile, Thr and Val compared to in-feed antibiotics (ABO) and multi-enzyme complex (ENZ) group. The improvement in growth performance and crude protein (CP) digestion of piglets fed with DFM can be attributed to several mechanisms. First, growth performance may be linked to nutrient utilization, as evidenced by the increased AID of CP and EAA in piglets fed DFM. Second, pigs fed diets containing DFM had a greater intestinal villus height, which may suggest improved digestive and absorptive capabilities. Third, metabolites from DFM can contribute to colonic volatile fatty acid (VFA) production and the regulation of systemic immune responses, contributing to energy salvage, the inhibition of opportunistic pathogens and protection against inflammation. Fourth, DFM modulate the activities of digestive enzyme and boost digestive capacity in weanling pigs, which may positively influence animal health.

**Abstract:**

The potentials of ABO replacer of ENZ and DFM on growth performance, AID, colonic VFAs, gut morphology, fecal score and diarrhea incidence were evaluated. We randomly assigned 120 piglets to four experimental diets that included: (1) control diet (CON), fed the basal ration; (2) ABO was added at 250 ppm of in-feed ABO; (3) ENZ was added at a rate of 3 kg/ton feed; (4) DFM was added with 50 × 10^6^ cfu/g of *Bacillus subtilis* and 2 × 10^6^ cfu/g of *Lactobacillus* spp. at a rate of 1.2 kg/ton feed. A complete randomized design used six pens per treatment with five pigs per pen. Pigs had ad libitum access to feed and water throughout the 6-week trial. Feed intake and BW were recorded on weeks 0, 2, 4 and 6, as well as fecal scores and diarrhea incidences (visually recorded and calculated). At weeks 2 and 4, a sub-sample of pigs (*n* = 6) was sacrificed for intestinal morphology, enzyme activity and VFAs. The results of the study demonstrated that DFM piglets showed increased final BW (3 kg) (*p* < 0.001) vs. CON. Likewise, ADG was positively affected by the incorporation of ABO, ENZ and DFM in the diets, with an average increase of 8 to 17% on ADG compared with CON (*p* < 0.001). The AID of gross energy, organic matter, CP and EAAs in piglets fed ENZ and DFM were significantly higher (*p* < 0.05) than those of CON and ABO at weeks 2 and 4. Inclusion of DFM increased intestinal morphology, enzymatic activities and propionic and butyric acid more than in pigs fed CON, ABO and ENZ (*p* < 0.05). The fecal score and diarrhea incidence generally decreased over time in pigs fed DFM (*p* < 0.05). These findings indicate that dietary supplementation with DFM has better effects at any period on growth performance, CP and AA digestibility and beneficially altered the intestinal health in weanling piglets.

## 1. Introduction

Early weaning is an important period for improving the efficiency of growth in piglets and economic profits. However, this period experiences several changes and stress factors, including diets, environment, society and exposure to harmful pathogens, such as enterotoxigenic *Escherichia coli* (ETEC), which directly affects intestinal health [1]. ETEC infection has been related to post-weaning diarrhea (PWD), resulting in dehydration, reduced weight gain, lowered feed intake and malnutrition [2,3]. Some studies have shown that intestinal morphology is affected during the weaning transition, characterized by decreased villous height and increased crypt depth [4,5,6]. Reductions in protein digestibility may increase the risk of post-weaning diarrhea since undigested proteins can be used by pathogenic bacteria, e.g., ETEC [7,8]. The dramatic shifts in the epithelial lining of the small intestine, such as villus atrophy and crypt hyperplasia, lead to a further decrease in the capacity for digestion and absorption of available nutrients. Moreover, little apparent ileal digestibility (AID) research is available for piglets. Piglets have a limited capacity for protein digestion and AA absorption [9], mainly in the first week post-weaning [10]. Historically, to resolve PWD complications, in-feed antibiotics such as colistin have been used as the most effective way to prevent PWD. However, with increased bacterial resistance to antibiotics and the prohibition of colistin use in livestock, other types of feed additives are applied in pig production systems to prevent PWD. 

Multi-species DFM with cell-wall deficient *lactobacillus* spp. (L-forms) was reported as potential probiotics [11], and a strain of *Bacillus subtilis* was initially isolated in the laboratory. DFMs are defined as products that “contain live naturally occurring microorganisms”. In general, these products are composed of one or more strains of bacteria. According to Sanders and Huis [12], multi-strain and multi-species DFMs are more effective than mono-strain DFMs due to the specific health effects based on genera or species. The addition of DFM in weanling pigs results in improved feed efficiency and enhanced apparent total tract digestibility of protein in weanling pigs [13,14,15,16]. DFMs have superior characteristics in terms of heat resistance of spores, inhibitory activity against pathogenic bacteria, antibiotic resistance, production of enzymatic digestion and biofilm formation [17,18]. These properties make them promising candidates for probiotics in feed additives. However, some previous studies failed to observe such positive effects on improvements in feed intake, ADG or BW gain when *Bacillus* was included in weaning diets [19,20]. Enzymes, e.g., protease, have also been studied as having the potential to improve post-weaning feed intake, promote intestinal health and improve nutrient digestibility [21]. 

Although the impact on digestion (or related parameters) may often be inconsistent. Antibiotics, enzymes and probiotics have different modes of action in the gastrointestinal tract (GIT). Therefore, an experiment was conducted to study the hypothesis that DFM may positively affect growth, gut health and nutrient utilization sufficiently to serve as a viable alternative to ABO and ENZ. The objective of the present study was to evaluate the use of potential antibiotic replacers ENZ and DFM on growth performance, AID, gut morphology, digestive enzyme activities, colonic VFAs, fecal score, bacterial composition and diarrhea incidence when fed to weaned piglets.

## 2. Materials and Methods

### 2.1. Supplement Preparation

The in-feed antibiotics included 100 ppm of chlortetracycline hydrochloride, 120 ppm of colistin sulfate and 30 ppm of tiamulin hydrogen fumarate used as a positive control group. The multi-enzyme complex provided 2000 IU/g of α-amylase, 5000 IU/g of β-glucanase, 3000 IU/g of protease and 4000 IU/g xylanase. The probiotic used in this study was a mixture of spray-dried spores of *Bacillus subtilis* and *Lactobacillus* spp. containing at least 5 × 10^7^ colony-forming units (cfu/g) of *Bacillus subtilis* and 2 × 10^6^ cfu/g of *Lactobacillus* spp. It was fed at a rate of 1.2 kg/ton feed. Both enzyme activity and probiotics enumeration were confirmed by analyzing the feed samples.

### 2.2. Animals, Experimental Design and Diets

A total of 120, 3 weeks of age, crossbred [(Landrace × Large White) × Duroc] weaned pigs were randomly assigned based on the stratification of sexes and body weight into 4 treatments, including CON, ABO, ENZ and DFM. A completely randomized design was used, with 6 pens per replication and 5 pigs per pen (3 gilts and 2 castrated barrows). 

All diets were provided in mash form, and feed additives were constantly added through premixtures by using soybean meal as carrier and diluent. The CON group was fed the basal ration. The ABO group was fed the basal ration supplemented with 120 ppm of in-feed ABO. The ENZ group was fed the basal ration supplemented with multi-enzyme complex added at a rate of 3 kg/ton feed. The DFM group was fed the basal ration supplemented with DFM added at a rate of 1.2 kg/ton feed.

Experimental diets were formulated into two phases, including early and late weaning diets, in which pigs were fed at 1 to 2 and 3 to 6 weeks, respectively. Ingredient and chemical compositions of the experimental diet are provided in Table 1. Both basal diets were formulated to meet the nutrient requirement recommendations according to the NRC [22]. The basal ration contained an anti-mycotoxin product (Mycofix Plus 5.0, Biomin GmbH, Herzogenburg, Austria; 2 kg/ton feed). Diets were sampled prior to the start of the trial and at 2 and 4 weeks of the trial and submitted for mycotoxin analysis by LC-MS/MS (Activation Laboratories, Ancaster, ON, Canada).

Weaning pens (1.5 m × 1.5 m) were in an evaporatively cooled building with nipple drinkers and a feeder. All diets were fed to pigs in mash form. All pigs were fed their experimental diets in a daily amount of 2 to 3 times the estimated energy requirement for maintenance. Piglets were allowed ad libitum access to feed and water. The 6-week study was conducted during the months of August–September, with temperature and relative humidity varying between 25.6 °C to 28.1 °C and 45% to 60%, respectively.

### 2.3. Data Recording and Sample Collection

Pigs were individually weighed at weeks 0, 2, 4 and 6. Average daily gain (ADG) was calculated on a pen basis from the individual BW of the pigs. Feed intake per pen was recorded by weighing feed added to the feeders and feed remaining at the end of each period to determine the average daily feed intake (ADFI) and the feed conversion ratio (FCR). At weeks 2 and 4 of the experiment, one barrow and gilt in each pen (total of 6 pigs per treatment; 3 males: 3 females) was randomly selected and euthanized for (1) intestinal morphology, (2) ileal digestive enzyme analysis and (3) colonic VFA determination.

### 2.4. Apparent Ileal Digestibility (AID)

Twenty-four barrows (six pigs/group) were surgically fitted with T-cannulas in the distal ileum [22]. After surgery, pigs were moved to individual pens (1.0 m × 0.5 m) in a temperature-controlled room. Daily feed allowance was adjusted to 2.8 times the maintenance requirement for DE (2.8 × 110 kcal of DE/kg of BW^0.75^; [23]).

Apparent ileal digestibility (AID) of nutrients and amino acids was conducted at weeks 2 and 4 by using Cr_2_O_3_ as the indigestible marker. Piglets were fed diets containing 0.3% of Cr_2_O_3_ for 7 days prior to the collection day. Approximately 10 g of ileal sample from each pig were placed in separate bags and stored at −80 °C to prevent bacterial AA degradation. Sub-samples were divided into 2 parts. 

First, gross energy (GE), dry matter (DM), organic matter (OM) and crude protein (CP) of feed and ileal digesta were measured according to the AOAC [24] methods. GE was determined by measuring the heat of combustion in the samples using a bomb calorimeter (C 6000; IKA-Werke GmbH & Co., Staufen, Germany). Dry matter content was measured by weighing sample in crucible and drying for an oven over night at 105 °C to reach constant weight (procedure 930.15, [24]) ash was determined after ignition of a known weight of diets and feces in a furnace (Nabertherm, Bremen, Germany) at 500 °C. (procedure 942.05; [24]), and N by using the Kjeldahl procedure with Kjeltec (Kjeltec TM 2200, Foss Tecator, Hilleroed, Denmark). These samples were analyzed for CP content (nitrogen × 6.25; procedure 988.05; [24]).

Second, samples were thawed and lyophilized (beta 1-8 LSCbasic freeze dryer, MARTIN CHRIST, GmbH, Germany). Amino acids (excluding tryptophan) were prepared using AdvanceBio Amino Acid Reagents Kit (AAA, Agilent, Santa Clara, CA, USA) and analyzed using HPLC (Poroshell HPH-C18 Column, Agilent, Santa Clara, CA, USA) after acid hydrolysis for 24 h in 6 M HCl. Amino acids in hydrolysates were determined by HPLC after post-column derivatization [25]. 

An atomic absorption spectrophotometry, AAS (Perkin Elmer 3110), was used to determine Cr_2_O_3_ with a Perkin Elmer lamp for Cr (part#303-6021 Serial H235571). Standard for AAS from J.T. Baker 1000 µg/mL CAS 6449-04 for chromium. 

Values for AID of nutrients and amino acids were calculated according to the method described by Stein et al. [26]. The coefficients of the AID were calculated from these data by using the following formula.

AID (%) = 1 × (nutrient in ileal content (g/kg DM)/nutrient in diet (g/kg DM)) × (Cr_2_O_3_ in diet (g/kg DM)/Cr_2_O_3_ in ileal content (g/kg DM)) × 100

### 2.5. Intestinal Morphology

Sampling of the small intestine followed a procedure previously described [27]. The small intestine was dissected in three 10 cm cross-sectional segments of the proximal duodenum, mid jejunum and distal ileum, rinsed in ice-cold phosphate-buffered saline, cut into 5 cm segments and fixed in 10% neutral buffered formalin. The fixed intestinal segments were prepared according to standard paraffin-embedding techniques and stained with hematoxylin and eosin (H & E).

Villous height and crypt depth were measured using a digitized board coupled to a video monitor (Olympus Polaroid DMC-IE camera, Polaroid Corp., Waltham, MA, USA). Output from the digitizing board was collected using the program SigmaScan Pro 5 (SPSS Inc., Chicago, IL, USA). The villous height (VH) was measured from the tip to the base, and crypt depth (CD) was measured from the base of the villus to the base of the crypt. The villous height to crypt depth ratio (VCR) was calculated.

### 2.6. Digestive Enzyme Analysis

The digesta sample from ileal was homogenized in ice-cold saline at the ratio of 1:9 (g/mL). The homogenate was centrifuged at 2500× *g* for 10 min (4 °C) to collect the supernatant and use it for determination. The activities of chymotrypsin (ab234051), trypsin (ab102531) (Abcam Inc., Cambridge, MA, USA), α-amylase (MBS 007373) (MyBioSource Inc., San Diego, CA, USA) and sucrase (E-BC-K751-M) (Elabscience, Houston, TX, USA) were measured by using commercial assay kits according to the manufacturer’s instructions.

### 2.7. Colonic Volatile Fatty Acid

Colonic VFAs were assayed by gas chromatography, according to Morlein et al. [28]. Briefly, 5 g of colon content was weighed into a 15 mL centrifuge tube, and 2 mL of ultrapure water was added. The contents were vortex mixed for 30 s, allowed to stand at 4 °C for 30 min, and then centrifuged at 4 °C (10,000× *g*) for 10 min. To 1 mL of the supernatant, 0.2 mL of 25% metaphosphoric acid (*w*/*v*) was added, mixed well, allowed to stand at 0 °C for more than 30 min and centrifuged at 4 °C (10,000× *g*) for 10 min. In total, 1 mL of the subsequent supernatant and 1 mL of internal standard (0.5 g 3-methyl-n-valeric acid in 1 liter of 0.15 mol/L oxalic acid) were mixed with 3 mL of distilled water. One milliliter of the supernatant was centrifuged at 10,000× *g* for 4 min at 4 °C. Then, 500 μL of the supernatant was taken into a sample bottle for measurement using gas chromatography with a flame ionization detector (GC-2010plus, Shimadzu Corporation, Kyoto, Japan). 

### 2.8. Fecal Microbial Examination, Consistency and Diarrhea Incidence

Fecal samples were collected directly from pigs at weeks 0, 2, 4 and 6 for determination of *coliforms* and *E. coli*. Immediately following the collection, samples were transported to Betagro laboratory (Betagro Science Center, Bangkok, Thailand) and stored at −80 °C until used. The reference method for fecal microbial examination was in accordance with AOAC 991.14 methods [25]. 

Fecal score was also evaluated at the same time of collection, which was visually assessed each morning by observers blinded to treatments using a modification of the method described by Liu et al. [29]. Fresh excreta were ranked using the following scale: 0 = solid; 1 = semi-solid; 2 = semi-liquid; and 3 = liquid.

Diarrhea incidence = (No. of pigs in each treatment group has a clinical sign of diarrhea on the same day × 100)/Total number of pigs in the treatment group. This equation was adapted from Hart and Dobb [30].

### 2.9. Statistical Analysis

The model used was *Yij* = *μ* + *Ti* + *Rj* + *eij*, where *Yij* is an observation on the dependent variable *ij*, *μ* is the overall population mean, *Ti* is the fixed effect of feed additive treatments, *Rj* is the pen as a random effect, and *eij* is the random error associated with the observation *ij*. All experimental data were analyzed by using the GLM procedure of SAS 9.0 as a completely randomized design (SAS Institute Inc., Cary, NC, USA). 

Statistical differences were considered significant at *p* ≤ 0.05 and highly significant at *p* < 0.01, and trends are suggested at *p* < 0.10. The pen was the experimental unit. Statistical differences among treatments were separated by Duncan’s multiple-range tests. Fecal microbial data were log-10 transformed to align measures to a normal distribution. The Chi-square test was used for analyzing the percentage of diarrhea incidence for an overall experimental period. The presence of outliers was tested by a Grubb’s test, and 21 outliers were excluded from further analyses, i.e., 11 piglets were excluded from the calculation of FCR between weeks 0–2 and 2–4 and 10 piglets from the calculation of FCR between weeks 4–6.

## 3. Results

### 3.1. Growth Performance

The effects on body weight, average daily gain, feed intake and feed efficiency of pig diets supplemented with ABO, ENZ and DFM are presented in Table 2. All pigs remained healthy throughout the experiment and readily consumed their daily feed allowances. In the whole period of the experiment, 2, 4 and 6 weeks, the DFM group achieved a final BW of 11.58, 17.07 and 22.98 kg, respectively, significantly higher than CON, ABO and ENZ (*p* < 0.001). In the first 2 weeks, the DFM group showed a significantly higher ADG compared to the other groups (*p* < 0.010), but no difference was found after 4 and 6 weeks. Supplementation with ENZ and DFM significantly increased the ADFI during weeks 2 and 4 (*p* < 0.001), and dietary with ABO and ENZ significantly decreased ADFI during weeks 4 to 6 (*p* < 0.001) compared to the CON group. No differences in FCR were found in the whole period, but there were differences in the mean FCR in the DFM group compared with that in CON and ENZ (*p* < 0.001).

### 3.2. Apparent Ileal Digestibility of Nutrient and Amino Acid

Table 3 shows the apparent ileal digestibility (AID) of gross energy (GE), dry matter (DM), organic matter (OM) and crude protein (CP) in weanling pigs supplemented with ABO, ENZ and DFM. The ABO, ENZ and DFM groups significantly increased AID of GE compared to the CON group in week 2 (*p* < 0.037). Pigs fed ENZ and DFM had significantly higher (*p* < 0.004) AID of CP compared to CON and ABO group in week 2. Pigs fed ENZ and DFM had significantly higher (*p* < 0.032) AID of OM compared to the CON group, and pigs fed DFM had significantly higher (*p* < 0.001) AID of CP compared to CON, ABO and ENZ groups in week 4. There were no differences in AID of DM and OM in week 2 (*p* > 0.05) and AID of GE and DM in week 4 (*p* > 0.05) among piglets fed CON, ABO, ENZ and DFM.

Table 4 shows the apparent ileal digestibility (AID) of essential amino acids (EAAs) and non-essential amino acids (nEAAs) in weanling pigs supplemented with ABO, ENZ and DFM.

The results, AID in week 2, indicated that AID of Arg (*p* < 0.014), Ile (*p* < 0.029), Val (*p* < 0.024), total EAAs (*p* < 0.004), Cys (*p* < 0.025), total nEAAs (*p* < 0.013) and total AAs (*p* < 0.001) was greater in the DFM group compared with the CON diet. The results, AID in week 4, indicated that AID of Arg (*p* < 0.003), Thr (*p* < 0.001), Val (*p* < 0.001), total EAAs (*p* < 0.001), Ala (*p* < 0.026), Cys (*p* < 0.014) and total AAs (*p* < 0.001) was greater in the DFM diet compared with the CON diet. There were no differences in AID (*p* > 0.05) of His, Leu, Lys, Phe, Thr, Ala, Asp, Glu, Gly, Pro, Ser and Tyr in week 2, and no differences in AID of His, Leu, Asp, Glu, Gly, Pro, Ser, Tyr and total nEAAs in week 4 among piglets fed CON, ABO, ENZ and DFM. 

### 3.3. Intestinal Morphology

**Figure 1:**
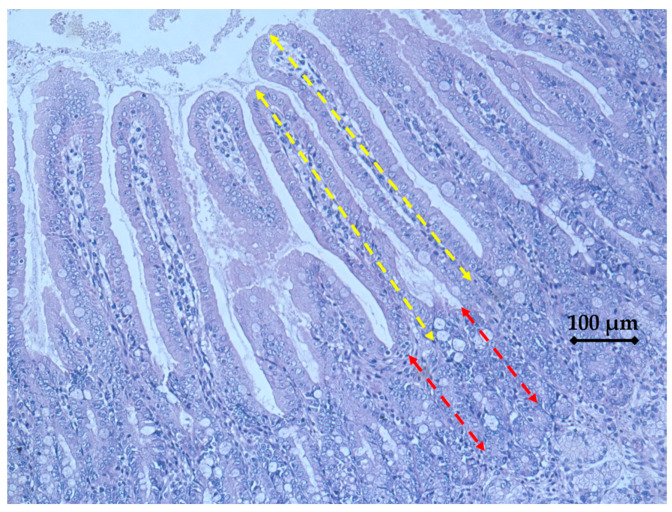
Morphometric measurements of villus height (yellow) and crypt depth (red) in weanling pigs. Representative image (200× magnification) of intestinal morphology and bar (100 μm).

**Figure 2:**
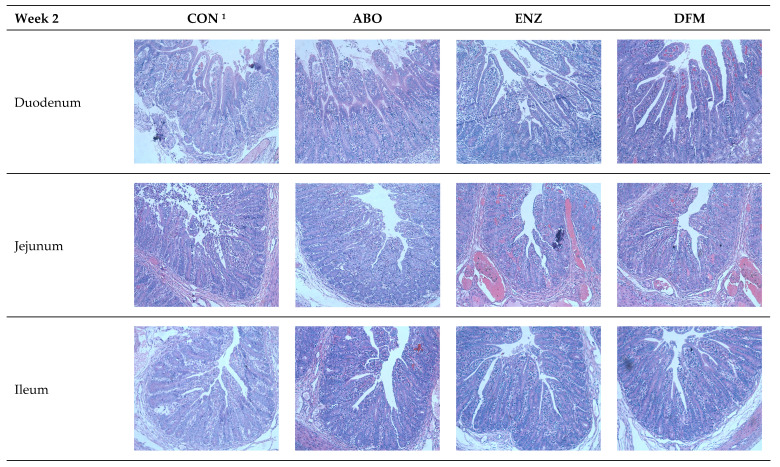

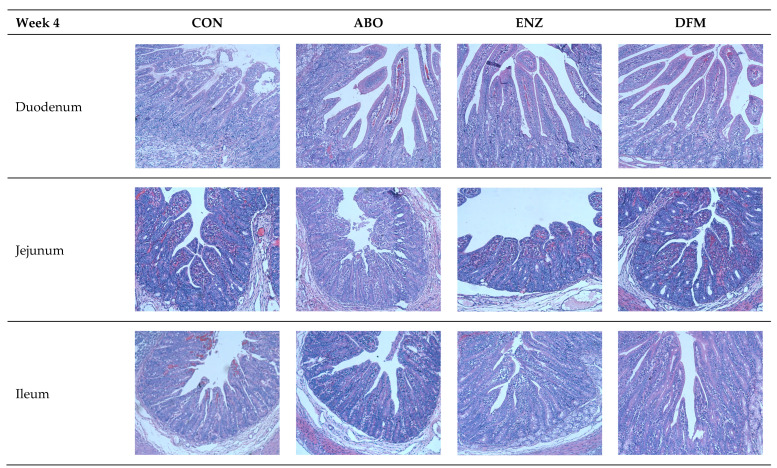
Effect of dietary treatment on intestinal morphology of weaning piglets in the duodenum (200× magnification), jejunum and ileum (100× magnification). ^1^ CON—control diet was the basal ration; ABO—the basal plus 100 ppm of chlortetracycline hydrochloride, 120 ppm of colistin sulfate and 30 ppm of tiamulin hydrogen fumarate; ENZ—the basal plus 2000 IU/g of α-amylase, 5000 IU/g of β-glucanase, 3000 IU/g of protease and 4000 U/g xylanase; DFM—the basal plus 5 × 10^7^ cfu/g *Bacillus subtilis* and 2 × 10^6^ *Lactobacillus* spp.

Figure 1 and Figure 2 and Table 5 show morphometric measurements, villous height (VH), crypt depth (CD) and their ratio (VCR) in small intestines of piglets post-weaning. From 0 through 2 weeks, piglets on DFM showed highly significant increases (*p* < 0.006) in villous height and villous crypt ratio in the ileum segment but not the jejunum or ileum. Similarly, VH increased (*p* < 0.01) throughout the intestine of the duodenum (*p* < 0.034), jejunum (*p* < 0.008) and ileum segments (*p* < 0.007) in week 4 feeding (*p* < 0.05). There are no significant differences in CD or VCR.

### 3.4. Ileal Digestive Enzyme Activities

The determination of ileal digestive enzyme activities on chymotrypsin, trypsin, amylase and sucrase is shown in Table 6. No statistically significant differences were observed regarding digestive enzyme activities in ileal digesta content in week 2 (*p* > 0.05). At week 4, compared with the CON, pigs fed DFM had greater (*p* < 0.05) enzyme activities of chymotrypsin (*p* < 0.007) and trypsin (*p* < 0.033). No differences were observed in enzyme activities of amylase and sucrase among piglets fed CON, ABO, ENZ and DFM (*p* > 0.05).

### 3.5. Colonic Volatile Fatty Acids

At week 2, the concentrations of total VFAs (*p* < 0.003) and butyric acid (*p* < 0.038) were significantly improved in DFM pigs compared to CON (Table 7). Similarly, at 4 weeks, total VFAs (*p* < 0.005), propionic acid (*p* < 0.023) and butyric acid (*p* < 0.027) again improved in DFM pigs compared to CON. Molar percentages of VFAs did not change throughout the experiment from weeks 2 to 4.

### 3.6. Fecal Microbial Examination

At week 2, fecal *coliforms* and *E. coli* were significantly decreased (*p* < 0.001) in ABO, ENZ and DFM compared to the CON group in week 2 (Table 8). In addition, the overall results of fecal *coliforms* and *E. coli* were significantly reduced (*p* < 0.0001) from weeks 4 to 6 in pigs fed ABO, ENZ and DFM compared to the CON group. This effect was sustained for the remainder of the experiment. 

### 3.7. Fecal Score and Diarrhea Incidence

**Figure 3:**
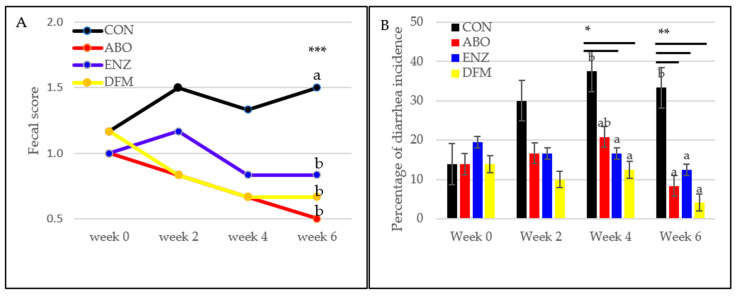
(**A**). Effect of dietary treatment on fecal scores of weaned piglets. Fresh excreta were ranked using the following scale: 0 = solid; 1 = semi-solid; 2 = semi-liquid; and 3 = liquid. Source: Liu et al. [29]; (*** *p* < 0.001). (**B**). Effect of dietary treatment on diarrhea incidence of weaned piglets. Diarrhea incidence = (No. of pigs in each treatment group has a clinical sign of diarrhea on the same day ×100)/Total number of pigs in the treatment group. Source: Adapted from Hart and Dobb [30]; (* *p* < 0.05, ** *p* < 0.01). ^a,b^ Different superscripts within a week indicate a significant difference. Abbreviation; CON—control diet was the basal ration; ABO—the basal plus 100 ppm of chlortetracycline hydrochloride, 120 ppm of colistin sulfate and 30 ppm of tiamulin hydrogen fumarate; ENZ—the basal plus 2000 IU/g of α-amylase, 5000 IU/g of β-glucanase, 3000 IU/g of protease and 4000 U/g xylanase; DFM—the basal plus 5 × 10^7^ cfu/g *Bacillus subtilis* and 2 × 10^6^ *Lactobacillus* spp.

Some CON pigs displayed mild diarrhea during the second week after weaning. Despite this, there were no significant differences in fecal score and diarrhea incidence between the treatments. Pigs that received ABO, ENZ and DFM had better fecal scores (*p* < 0.001) compared to the CON group in week 6 (Figure 3A). Percentage of diarrhea incidence (Figure 3B) was high in the CON group compared to the ENZ and DFM groups at week 4 (*p* < 0.05). This was also found when compared to ABO, ENZ and DFM at week 6 (*p* < 0.01).

## 4. Discussion

In commercial swine production, early weaning ages range from 3–4 weeks of age in order to increase economic efficiency. Consequently, impairment of digestive functions, e.g., insufficient secretion of digestive enzymes, results in significantly poor feed intake, low feed utilization and slow growth [1,2,3,4,5,6]. Our results indicated that DFM supplementation in weanling pigs significantly increased growth, nutrient digestibility, intestinal health and enzymatic activities while the incidence of diarrhea decreased throughout the whole experiment. It has been proposed that multi-species DFMs can be more effective due to the combination of different modes of action of various genera. Many DFMs have been shown to be beneficial in animal production. They are used to mitigate pathogens [31], provide extracellular enzymes [18], release antimicrobial compounds [32] and other actions, including for *Bacillus* spp., increase apparent ileal digestibility in weanling pigs [13,14,15,16]. 

The present data demonstrate evidence that DFM was providing essential nutrient support in the early weaning period. Advantages in body weight gain (BWG) were variable by week but overall showed an improvement in DFM pigs. Daily gains, too, showed weekly variation; however, weekly improvements in ADG ranged from 8 to 17%, with an overall 20% better growth for the trial. During phase 2 (weeks 2–6 post-weaning), pigs fed DFM had significant growth rates than those fed ABO. However, the results of the current study contradict those reported in the study by Walsh et al. [33], who found the BWG increase in an ABO group was better than in DFM. Moreover, weight data are, in part, commensurate with improved feed intakes, while FCR, except for weeks 2, 4 and 6, was not significant. Three possible reasons for these changes are apparent. A factor yet unrecognized in DFM acted as a prebiotic or an appetite stimulant, increasing intake and gains in the treated piglets. Second, it is possible that some discrepancies in *Bacillus*’ effect on pig growth are caused by a variety of factors, including diet formula, different *Bacillus* species, dose, pig age or environmental factors [34]. Finally, the presence of observable effects of antibiotic feeding on growth during the first 2 weeks post-weaning in the field study may be attributed to the synergy between chlortetracycline [35] and tiamulin [36].

Dietary supplementation with DFM resulted in increased AID of GE, OM, CP and AAs for 2 and 4 weeks after weaning, while no effects were found in DM. Overall, CP digestibility was significantly increased by supplemental DFM and ENZ, which agrees with other studies [13,14,37]. It is well demonstrated that *Bacillus subtilis* is capable of producing and secreting various enzymes and is a superior enzyme producer compared to other strains of *Bacillus.* This may explain the clear positive effects of the DFM for digestibility in the present study. Blavi et al. [38] showed that the contrast result in AID of CP was significantly lower in pigs fed a single strain of *B. subtilis* compared to CON diets. It is possible that *B. subtilis* may synthesize alter enzymes that hydrolyze fibers, such as peptide and xylanase [39], thereby contributing to increased fiber fermentation and subsequent production and absorption of VFAs. A single strain of DFM in previous research might have been ineffective to be a digestive enhancer. Hence, Cai et al. [13] demonstrated double strain DFMs comprised of *B. subtilis* and *B. amyloliquefaciens*, when fed to weanling pigs, resulted in increased apparent total-tract digestibility (ATTD) of CP digestibility compared to CON diet. Arg, Ile, Thr and Val are of interest since, for example, supplementing the former has been shown to improve the growth performance of modern swine breeds [40]. Arg plays various synthetic precursors of physiological functions, especially in protein synthesis and antioxidant ability [41]. Ile and Val are branched-chain AAs (BCAAs), and supplementation of BCAAs has been related to stimulated muscle growth and feed intake [42]. Thr is the second limiting amino acid for swine and has important roles in maintaining intestinal mucosal integrity and barrier function in piglets [43]. Cys was significantly increased by nearly 10% above the control, and Met rose as well. The role of Cys as an endogenous antioxidant [40] and Met’s action in enhancing intestinal morphology, reducing oxidative stress and improving glutathione production [8] give additional emphasis to the positive effects of feeding this DFM.

In the present study, feeding DFM produced consistently taller villi in the duodenum, jejunum and ileum during both phases of feeding. VCR was also improved, although more variably. The notable exception was in phase 2 for the jejunum. In this study, piglets fed the DFM diet had longer villi than the CON, ABO and ENZ, which explains improved growth and digestibility in the present study. Wang et al. [43] showed that VH is positively associated with the small intestinal digestive enzyme activity and expression of nutrient transporters. The growth performance of DFM piglets is better than those of CON, ABO and ENZ piglets, which may be due to better nutrient digestion and absorption. In agreement with our results, Lee et al. [44] reported linear effects of *B. subtilis* on VH and VCR, which are generally associated with better nutrient utilization. However, some authors found that villi integrity was not affected by DFM compared to CON. They suggested this might arise from an immune response to a dietary antigen and lack of luminal stimulation from reduced feed intake during the early weaning period [45,46]. Considering the present data, the DFM used here had a positive effect on intestinal architecture and, when coupled with fecal scores and diarrheal incidence, was successful in controlling diarrhea.

From this study, the results indicated that high enzyme activities of chymotrypsin and trypsin are a direct result of DFM inclusion compared to CON and ABO. Pancreatic enzymes, such as trypsin and chymotrypsin, digest protein into peptides and free AAs [25]. In contrast, there were no significant differences between treatments on enzyme activity in the early weaning period. Several studies have reported equivocal effects of weaning on gastric enzyme activity. For example, feeding SBM in the weanling’s diet could inhibit chymotrypsin activities in jejunal digesta, which are associated with the incidence of diarrhea and the appearance of ETEC in piglets [47]. There is clear evidence in the literature that excessive amounts of undigested protein will be fermented in the large intestine and can be utilized by pathogenic bacteria, causing PWD [7,8]. Activities of the digestive enzymes, such as pepsin, trypsin, chymotrypsin and amylase, were influenced mainly by age and decreased significantly within one week of early weaning, resulting in disturbance of the immature digestive system [48].

Blavi et al. [38] showed significant hind gut CP digestibility, which provided essential nutrients to colonic VFA fermentation. Similarly, feeding DFM produced highly significant responses in colonic volatile fatty acids. When *Bacillus*-based DFMs germinate in the intestine of the pig, they may produce a wide variety of fiber-degrading enzymes [48]. Therefore, the addition of a *Bacillus*-based DFM may enhance the fermentation of dietary fiber in swine diets and, subsequently, increase the available energy from the diet in the form of VFAs [49]. During both phase 1 and 2 feeding, specific VFAs increased, and the distribution of these was improved (e.g., reduction in acetate to propionate ratio). Butyrate increased by 80% and 35% at D14 and D28, respectively. Short-chain fatty acids and low digesta pH are known to support the growth of beneficial bacteria at the expense of *Enterobacteria* spp. [50]. With acetate and propionate supporting peripheral tissue [51] and butyrate supplying energy for colonic cells, inhibiting inflammation and carcinogenesis, reinforcing the colonic barrier defense and decreasing oxidative stress [52], the reasonable expectation that our DFM would produce such effects and enhance the VFA production was clearly shown by the present data.

Fecal microbial data would give greater credence to the latter two thoughts on DFM actions. Total *coliforms* and *E. coli* were reduced by 2–3 logs over the six-week study. The single exception to the magnitude of this change occurred in week 4 when only a 1 log decrease was observed, although, even then, the reduction was highly significant. These piglets were phase-fed, and the fourth-week measurement could reflect an effect of change in ration. That change is major for young pigs, which generally exhibit transient growth stasis, some reductions in digestion/absorption and a proliferation of pathogenic bacteria [45,46]. Regardless, the reductions in these bacteria by DFM are more likely the result of mitigated ETEC challenge (which can suppress GIT immune function) or the probiotic effects of DFM promoting GIT immunity and microbiome development. 

Further to this point are the fecal microbial data. Enterotoxins from ETEC are the main cause of the diarrhea incidence as found in piglets on the CON diet. One factor involved is undigested protein, which increases microbial metabolites in colonic digesta and aggravates piglets’ diarrhea [8]. Based on our study, CP digestion was low in the CON group, and those piglets showed higher incidences of diarrhea throughout the study. The reduction of fecal score in piglets fed DFM has been explained by Zhao and Kim [53]. In their work, dietary supplementation of a *Lactobacillus* complex inhibited the adhesion of *E. coli* to the gastrointestinal mucosa, thus reducing the incidence of watery digesta and diarrhea. Differences in the ENZ and DFM treatments may be a result of the level of protease enzyme, which, by acting on undigested protein, potentially decreases the risk of post-weaning diarrhea [54]. Finally, an improved fecal score with lower diarrhea incidence in piglets fed diets supplemented with DFM suggests a healthy piglet with a balanced gut microbiome efficiently producing antibacterial substances, competitively excluding pathogens, and improving the mucosal barrier to prevent PWD.

## 5. Conclusions

Under commercial swine production conditions prevalent in Thailand, a specific DFM containing cell-wall deficient (L-form) *Lactobacillus* spp. and *B. subtilis* was tested against control, antibiotic and enzyme treatments for the prevention of post-weaning diarrhea. The DFM was found to be superior to antibiotics in all metrics tested; it performed at least as well as enzymes in a few variables but was significantly better than them in most. The data indicate improvements in feed intake and growth, nutrient digestibility, architecture of the small intestine and appropriate production of colonic volatile fatty acids. While further investigations in piglets, such as specific actions of DFM on crude protein in the weanling’s diet, are of interest, the strong positive effects also suggest a need to study the responses in the sow. The use of the DFM in this study strongly suggests it may be a useful alternative to other approaches to PWD as well as for improvements in the nutrition and management of commercial swine.

## Figures and Tables

**Table 1:** Ingredient composition and nutrient analysis of the basal diet (as fed).

Item	Basal Diets	
	Phase I	Phase II
Ingredient, %		
Broken rice	57.5	38
Soy bean meal 44%	21	25
Full-fat soy bean	10	14
Cassava meal		10
Rice bran oil	1.5	3
Prelac (milk replacer)	5	5
M-dicalcium phosphate	2.5	2.5
Calcium carbonate	1.4	1.4
L-Lysine HCl	0.3	0.3
L-Threonine	0.3	0.3
Vitamin/mineral premix ^1^	0.3	0.3
DL-methionine	0.1	0.1
Salt	0.1	0.1
Total	100	100
Analyzed nutrient levels, %		
ME (kcal/kg)	3480	3270
Dry matter	88.2	89.4
Crude protein	23	21.3
Starch	50.3	43.2
Crude fat	5.8	4.2
NDF	8.1	7.6
ADF	4.4	3.6
Essential Amino Acid, %		
Arginine	1.61	1.49
Histidine	0.56	0.53
Isoleucine	0.99	0.93
Leucine	1.66	1.54
Lysine	1.71	1.63
Methionine	0.45	0.43
Phenylalanine	1.1	1.02
Threonine	1.13	1.08
Tryptophan	0.31	0.28
Valine	1.09	1.01

^1^ The premix provided the per gram of diet as follows: 2.40 IU vitamin A, 0.48 IU vitamin D, 4000 IU vitamin E, 0.72 g vitamin K, 0.069 g vitamin B1, 1.04 g vitamin B2, 0.12 g vitamin B6, 0.006 g vitamin B12, 7.20 g nicotinic acid, 2.72 g pantothenic acid, 34.88 g Cu as copper sulfate, 20 g of Fe as ferrous sulfate, 0.23 g I as calcium iodate, 13.64 g Mn as manganese sulfate, 24 g Zn as zinc sulfate and 0.02 g Se as sodium selenite.

**Table 2:** Effect of dietary treatment on growth performance of weanling pigs ^1^.

Week	CON ^2^	ABO	ENZ	DFM	*p*-Value
Body Wt., kg					
0	6.09 ± 0.86	6.03 ± 0.71	5.83 ± 0.72	5.93 ± 0.83	0.560
2	10.32 ± 1.35 ^b^	11.00 ± 1.05 ^ab^	10.80 ± 1.81 ^ab^	11.58 ± 2.28 ^b^	0.030
4	14.81 ± 1.21 ^c^	16.08 ± 2.07 ^b^	16.30 ± 1.91 ^ab^	17.07 ± 1.40 ^a^	0.001
6	20.10 ± 1.66 ^c^	21.27 ± 2.06 ^b^	21.89 ± 2.61 ^ab^	22.98 ± 2.31 ^a^	0.001
ADG, g/d					
0–2	302 ± 119 ^b^	355 ± 81 ^ab^	355 ± 130 ^ab^	403 ± 162 ^a^	0.010
2–4	320 ± 112	362 ± 153	392 ± 139	392 ± 148	0.163
4–6	378 ± 94	370 ± 103	399 ± 129	421 ± 147	0.333
Mean ADG	333 ± 48 ^c^	362 ± 54 ^b^	382 ± 66 ^ab^	405 ± 57 ^a^	0.001
ADFI, g/d					
0–2	513 ± 21 ^c^	578 ± 48 ^b^	684 ± 61 ^a^	580 ± 27 ^b^	0.001
2–4	769 ± 33 ^b^	792 ± 55 ^b^	849 ± 40 ^a^	836 ± 61 ^a^	0.001
4–6	988 ± 124 ^a^	830 ± 37 ^c^	870 ± 39 ^bc^	886 ± 158 ^b^	0.001
Mean ADFI	757 ± 47 ^b^	733 ± 32 ^c^	801 ± 34 ^a^	767 ± 49 ^b^	0.001
FCR					
0–2	1.67 ± 0.44	1.66 ± 0.36	1.64 ± 0.41	1.40 ± 0.43	0.151
2–4	1.72 ± 0.38	1.68 ± 0.31	1.68 ± 0.30	1.72 ± 0.29	0.966
4–6	1.72 ± 0.46	1.69 ± 0.41	1.68 ± 0.35	1.65 ± 0.39	0.918
Mean FCR	1.69 ± 0.41	1.67 ± 0.23	1.65 ± 0.28	1.59 ± 0.34	0.127

^a–c^ Different superscripts within a row indicate a significant difference (*p* < 0.05). ^1^ The pen was considered as the experimental unit, with six replicate pens per treatment and five and four pigs per replicate pen in weeks 2 and 4, respectively. ^2^ CON—control diet was the basal ration; ABO—the basal plus 100 ppm of chlortetracycline hydrochloride, 120 ppm of colistin sulfate and 30 ppm of tiamulin hydrogen fumarate; ENZ—the basal plus 2000 IU/g of α-amylase, 5000 IU/g of β-glucanase, 3000 IU/g of protease and 4000 U/g xylanase; DFM—the basal plus 5 × 10^7^ cfu/g *Bacillus subtilis* and 2 × 10^6^ *Lactobacillus* spp.

**Table 3:** Effect of dietary treatment on apparent ileal digestibility (AID) of gross energy (GE), dry matter (DM), organic matter (OM) and crude protein (CP) in weanling pigs at 2- and 4-weeks post-weaning ^1^.

AID, %	CON ^2^	ABO	ENZ	DFM	*p*-Value
Week 2					
GE, %	78.96 ± 3.73 ^b^	81.10 ± 1.65 ^ab^	84.06 ± 1.90 ^a^	81.34 ± 2.07 ^ab^	0.037
DM, %	76.76 ± 4.06	77.96 ± 3.68	78.64 ± 3.33	80.56 ± 1.41	0.358
OM, %	84.08 ± 1.57	83.63 ± 1.02	83.19 ± 2.39	83.38 ± 2.26	0.852
CP, %	77.63 ± 2.01 ^c^	78.72 ± 2.07 ^bc^	80.51 ± 2.38 ^ab^	82.94 ± 1.60 ^a^	0.004
Week 4					
GE, %	80.11 ± 3.52	81.42 ± 2.34	83.27 ± 3.59	82.76 ± 3.01	0.414
DM, %	79.03 ± 3.74	77.86 ± 1.69	78.49 ± 3.73	80.37 ± 2.83	0.555
OM, %	81.09 ± 1.82 ^b^	82.93 ± 1.56 ^ab^	82.69 ± 1.96 ^ab^	83.95 ± 1.72 ^a^	0.032
CP, %	77.71 ± 1.82 ^c^	78.91 ± 2.08 ^c^	81.89 ± 1.17 ^b^	84.87 ± 0.77 ^a^	0.001

^a–c^ Different superscripts within a row indicate a significant difference (*p* < 0.05). ^1^ The pen was considered as the experimental unit, with six replicate pens per treatment and five and four pigs per replicate pen in weeks 2 and 4, respectively. ^2^ CON—control diet was the basal ration; ABO—the basal plus 100 ppm of chlortetracycline hydrochloride, 120 ppm of colistin sulfate and 30 ppm of tiamulin hydrogen fumarate; ENZ—the basal plus 2000 IU/g of α-amylase, 5000 IU/g of β-glucanase, 3000 IU/g of protease and 4000 U/g xylanase; DFM—the basal plus 5 × 10^7^ cfu/g *Bacillus subtilis* and 2 × 10^6^ *Lactobacillus* spp.

**Table 4:** Effect of dietary treatment on apparent ileal digestibility (AID) of amino acids in weanling pigs at 2- and 4-weeks post-weaning ^1^.

	CON ^2^	ABO	ENZ	DFM	*p*-Value
Week 2					
Arginine	79.46 ± 2.47 ^b^	81.73 ± 0.84 ^a^	81.16 ± 0.71 ^ab^	82.72 ± 0.86 ^a^	0.014
Histidine	72.93 ± 4.01	75.98 ± 6.21	71.47 ± 5.34	73.24 ± 6.72	0.592
Isoleucine	79.94 ± 2.09 ^ab^	78.28 ± 2.69 ^b^	80.95 ± 1.36 ^a^	81.67 ± 1.84 ^a^	0.029
Leucine	75.63 ± 4.03	78.63 ± 4.93	79.41 ± 3.02	81.36 ± 3.26	0.127
Lysine	77.25 ± 1.20	76.87 ± 4.32	75.89 ± 3.57	75.36 ± 3.46	0.777
Methionine	75.76 ± 3.98 ^b^	81.63 ± 2.32 ^a^	79.09 ± 4.05 ^ab^	76.07 ± 3.19 ^b^	0.008
Phenylalanine	82.56 ± 2.44	82.52 ± 2.69	82.95 ± 1.79	84.07 ± 1.64	0.623
Threonine	76.89 ± 1.53	76.43 ± 1.26	77.85 ± 1.98	79.19 ± 1.56	0.065
Valine	77.81 ± 1.80 ^b^	78.30 ± 1.98 ^b^	77.88 ± 3.15 ^b^	82.04 ± 2.72 ^a^	0.024
Total EAAs	77.58 ± 1.18 ^c^	78.93 ± 0.48 ^ab^	78.52 ± 1.02 ^bc^	79.52 ± 1.05 ^a^	0.004
Alanine	80.04 ± 1.99	81.24 ± 1.05	80.95 ± 0.84	83.58 ± 0.90	0.447
Aspartic acid	72.81 ± 4.42	75.75 ± 4.70	71.83 ± 3.81	73.11 ± 5.72	0.552
Cysteine	70.82 ± 4.27 ^b^	75.81 ± 3.58 ^ab^	73.75 ± 6.05 ^ab^	79.48 ± 5.70 ^a^	0.025
Glutamic acid	71.14 ± 4.53	76.69 ± 3.71	72.61 ± 4.50	72.82 ± 6.04	0.281
Glycine	72.13 ± 4.13	72.64 ± 4.81	72.32 ± 5.79	73.69 ± 6.78	0.963
Proline	71.17 ± 3.68	71.60 ± 3.69	73.98 ± 2.36	74.38 ± 3.92	0.325
Serine	71.04 ± 6.21	75.14 ± 5.01	72.05 ± 3.39	70.55 ± 5.64	0.394
Tyrosine	69.34 ± 3.20	71.38 ± 5.50	70.05 ± 5.07	74.49 ± 3.92	0.272
Total nEAAs	71.34 ± 1.15 ^c^	74.03 ± 0.85 ^a^	72.38 ± 1.37 ^bc^	73.48 ± 1.82 ^ab^	0.013
Total AAs	74.65 ± 0.54 ^c^	76.62 ± 0.51 ^a^	75.63 ± 0.92 ^b^	76.68 ± 1.35 ^a^	0.001
Week 4					
Arginine	80.04 ± 1.99 ^b^	81.24 ± 1.05 ^b^	80.95 ± 0.84 ^b^	83.58 ± 0.90 ^a^	0.003
Histidine	73.64 ± 3.85	73.91 ± 5.62	74.62 ± 5.46	78.20 ± 5.46	0.314
Isoleucine	79.36 ± 2.65	78.39 ± 1.53	80.65 ± 2.25	81.71 ± 1.74	0.093
Leucine	77.32 ± 3.93	76.61 ± 4.49	80.13 ± 2.27	78.17 ± 2.58	0.374
Lysine	78.93 ± 3.11	79.14 ± 2.72	80.41 ± 1.64	80.96 ± 1.94	0.175
Methionine	77.11 ± 2.97	83.04 ± 3.07	79.93 ± 3.33	79.20 ± 5.19	0.087
Phenylalanine	81.88 ± 1.85	83.76 ± 0.79	82.25 ± 1.83	81.21 ± 0.98	0.066
Threonine	75.24 ± 1.01 ^c^	76.48 ± 1.16 ^bc^	77.93 ± 2.02 ^ab^	78.95 ± 1.19 ^a^	0.001
Valine	78.27 ± 1.98 ^b^	77.98 ± 2.33 ^b^	79.13 ± 2.84 ^b^	82.78 ± 1.15 ^a^	0.001
Total EAAs	77.98 ± 1.09 ^c^	78.95 ± 0.84 ^bc^	79.55 ± 1.21 ^ab^	80.53 ± 0.81 ^a^	0.001
Alanine	69.45 ± 3.09 ^b^	72.62 ± 3.61 ^ab^	72.41 ± 2.95 ^ab^	75.64 ± 2.83 ^a^	0.026
Aspartic acid	71.74 ± 6.04	73.76 ± 5.39	72.29 ± 6.13	72.37 ± 5.45	0.935
Cysteine	74.04 ± 5.23 ^ab^	76.68 ± 7.41 ^ab^	71.93 ± 4.16 ^b^	80.35 ± 4.31 ^a^	0.014
Glutamic acid	70.60 ± 4.58	73.91 ± 4.43	74.51 ± 4.04	75.44 ± 2.46	0.265
Glycine	71.20 ± 3.70	73.12 ± 4.90	72.07 ± 3.90	74.19 ± 3.63	0.646
Proline	71.23 ± 3.33	69.87 ± 4.80	69.09 ± 4.80	72.21 ± 3.16	0.497
Serine	73.60 ± 5.43	71.52 ± 4.39	75.54 ± 3.70	75.29 ± 2.43	0.141
Tyrosine	70.50 ± 4.37	71.46 ± 4.39	72.61 ± 4.94	74.94 ± 4.51	0.467
Total nEAAs	72.34 ± 4.83	72.07 ± 5.73	73.98 ± 4.92	71.87 ± 4.76	0.872
Total AAs	74.95 ± 1.17 ^c^	76.09 ± 0.90 ^bc^	76.26 ± 0.72 ^b^	77.95 ± 0.76 ^a^	0.001

^a–c^ Different superscripts within a row indicate a significant difference (*p* < 0.05). ^1^ The pen was considered as the experimental unit, with six replicate pens per treatment and five and four pigs per replicate pen in weeks 2 and 4, respectively. ^2^ CON—control diet was the basal ration; ABO—the basal plus 100 ppm of chlortetracycline hydrochloride, 120 ppm of colistin sulfate, and 30 ppm of tiamulin hydrogen fumarate; ENZ—the basal plus 2000 IU/g of α-amylase, 5000 IU/g of β-glucanase, 3000 IU/g of protease and 4000 U/g xylanase; DFM—the basal plus 5 × 10^7^ cfu/g *Bacillus subtilis* and 2 × 10^6^ *Lactobacillus* spp.

**Table 5:** Effect of dietary treatment on villus height (VH), crypt depth (CD) and its ratio (VCR) in weanling pigs at 2- and 4-weeks post-weaning ^1^.

	Metric	CON ^2^	ABO	ENZ	DFM	*p*-Value
Week 2	µm					
Duodenum	VH	394 ± 63	441 ± 75	494 ± 86	441 ± 121	0.064
	CD	249 ± 28	253 ± 33	261 ± 23	262 ± 30	0.845
	VCR	1.60 ± 0.30	1.76 ± 0.30	1.88 ± 0.27	1.74 ± 0.67	0.416
Jejunum	VH	358 ± 89	371 ± 107	406 ± 88	533 ± 41	0.392
	CD	248 ± 61	182 ± 52	203 ± 42	224 ± 73	0.872
	VCR	1.52 ± 0.51	2.25 ± 1.10	2.07 ± 0.54	2.69 ± 1.18	0.693
Ileum	VH	424 ± 75 ^b^	494 ± 88 ^b^	475 ± 36 ^b^	578 ± 60 ^a^	0.006
	CD	215 ± 73	200 ± 63	204 ± 60	245 ± 41	0.254
	VCR	2.22 ± 1.04	2.60 ± 0.58	2.49 ± 0.69	2.41 ± 0.43	0.173
Week 4	µm					
Duodenum	VH	541 ± 59 ^b^	683 ± 86 ^a^	666 ± 117 ^a^	713 ± 82 ^a^	0.034
	CD	340 ± 108	332 ± 84	311 ± 76	350 ± 89	0.912
	VCR	1.72 ± 0.55	2.23 ± 0.88	2.30 ± 0.89	2.13 ± 0.50	0.617
Jejunum	VH	579 ± 70 ^b^	655 ± 92 ^b^	703 ± 85 ^b^	714 ± 116 ^a^	0.008
	CD	350 ± 103	347 ± 84	310 ± 74	355 ± 91	0.542
	VCR	1.81 ± 0.70	1.95 ± 0.39	2.39 ± 0.71	2.08 ± 0.44	0.831
Ileum	VH	537 ± 58 ^b^	552 ± 43 ^b^	585 ± 75 ^b^	678 ± 43 ^a^	0. 007
	CD	258 ± 36	274 ± 49	259 ± 24	257 ± 42	0.813
	VCR	1.71 ± 0.21	1.70 ± 0.39	1.89 ± 0.39	2.02 ± 0.37	0.280

^a,b^ Different superscripts within a row indicate a significant difference (*p* < 0.05). ^1^ The pen was considered as the experimental unit, with six replicate pens per treatment and five and four pigs per replicate pen in weeks 2 and 4, respectively. ^2^ CON—control diet was the basal ration; ABO—the basal plus 100 ppm of chlortetracycline hydrochloride, 120 ppm of colistin sulfate and 30 ppm of tiamulin hydrogen fumarate; ENZ—the basal plus 2000 IU/g of α-amylase, 5000 IU/g of β-glucanase, 3000 IU/g of protease and 4000 U/g xylanase; DFM—the basal plus 5 × 10^7^ cfu/g *Bacillus subtilis* and 2 × 10^6^ *Lactobacillus* spp.

**Table 6:** Effect of dietary treatment on activities of ileal digestive enzyme in weanling pigs at 2- and 4-weeks post-weaning ^1^.

	CON ^2^	ABO	ENZ	DFM	*p*-Value
Week 2					
Chymotrypsin (×10^3^ U/mg prot)	41.69 ± 5.66	42.56 ± 5.00	45.55 ± 4.49	49.18 ± 7.54	0.138
Trypsin (×10^3^ U/mg prot)	34.62 ± 2.66	34.49 ± 3.97	37.08 ± 2.53	37.82 ± 1.55	0.121
Amylase (U/mg prot)	2.52 ± 1.07	2.59 ± 0.89	3.10 ± 0.37	2.36 ± 0.69	0.492
Sucrase (U/mg prot)	168 ± 27	154 ± 31	135 ± 35	144 ± 29	0.286
Week 4					
Chymotrypsin (×10^3^ U/mg prot)	37.56 ± 2.34 ^b^	43.82 ± 8.36 ^ab^	48.51 ± 9.27 ^a^	51.65 ± 2.86 ^a^	0.007
Trypsin (×10^3^ U/mg prot)	35.76 ± 1.63 ^b^	36.73 ± 1.32 ^b^	36.21 ± 1.64 ^b^	39.91 ± 4.11 ^a^	0.033
Amylase (U/mg prot)	3.69 ± 0.93	3.45 ± 1.21	4.05 ± 0.42	3.37 ± 0.97	0.589
Sucrase (U/mg prot)	172 ± 25	159 ± 22	166 ± 21	148 ± 33	0.362

^a,b^ Different superscripts within a row indicate a significant difference (*p* < 0.05). ^1^ The pen was considered as the experimental unit, with six replicate pens per treatment and five and four pigs per replicate pen in weeks 2 and 4, respectively. ^2^ CON—control diet was the basal ration; ABO—the basal plus 100 ppm of chlortetracycline hydrochloride, 120 ppm of colistin sulfate and 30 ppm of tiamulin hydrogen fumarate; ENZ—the basal plus 2000 IU/g of α-amylase, 5000 IU/g of β-glucanase, 3000 IU/g of protease and 4000 U/g xylanase; DFM—the basal plus 5 × 10^7^ cfu/g *Bacillus subtilis* and 2 × 10^6^ *Lactobacillus* spp.

**Table 7:** Effect of dietary treatment on total and molar ratios of VFAs (mmol/g fresh sample) in colon in weanling pigs at 2- and 4-weeks post-weaning ^1^.

	CON ^2^	ABO	ENZ	DFM	*p*-Value
Week 2					
Total VFAs (mmol/g fresh sample)	96.67 ± 10.14 ^b^	123 ± 10 ^a^	118 ± 14 ^a^	126 ± 12 ^a^	0.003
Acetic acid	53.27 ± 8.33	63.27 ± 9.06	56.83 ± 13.93	60.80 ± 11.70	0.434
Propionic acid	32.30 ± 4.97	45.27 ± 10.35	46.70 ± 13.64	47.43 ± 5.90	0.073
Butyric acid	11.10 ± 3.07 ^b^	15.40 ± 3.20 ^ab^	15.40 ± 3.32 ^ab^	18.00 ± 3.50 ^a^	0.038
A:P ratio	1.69 ± 0.41	1.49 ± 0.56	1.36 ± 0.67	1.30 ± 0.33	0.626
Percentage					
Acetic acid, %	55.10 ± 6.06	51.19 ± 6.94	47.95 ± 10.59	47.93 ± 6.04	0.387
Propionic acid, %	33.48 ± 4.17	36.43 ± 7.04	39.15 ± 9.69	37.79 ± 4.94	0.584
Butyric acid, %	11.42 ± 2.59	12.38 ± 2.10	12.90 ± 2.14	14.28 ± 2.57	0.304
Week 4					
Total VFAs (mmol/g fresh sample)	142 ± 19 ^c^	168 ± 13 ^b^	190 ± 18 ^ab^	197 ± 26 ^a^	0.005
Acetic acid	85.57 ± 16.56	95.70 ± 17.78	111.77 ± 14.78	108.93 ± 25.19	0.092
Propionic acid	40.13 ± 8.26 ^b^	48.10 ± 9.68 ^ab^	56.63 ± 5.20 ^a^	56.40 ± 13.35 ^a^	0.023
Butyric acid	16.57 ± 4.58 ^b^	24.73 ± 7.50 ^ab^	22.37 ± 7.59 ^ab^	32.27 ± 8.53 ^a^	0.027
A:P ratio	2.19 ± 0.51	2.06 ± 0.54	1.98 ± 0.25	2.06 ± 0.72	0.933
Percentage					
Acetic acid, %	59.90 ± 6.59	56.46 ± 6.91	58.51 ± 3.27	54.66 ± 8.83	0.677
Propionic acid, %	28.23 ± 4.56	28.55 ± 5.57	29.81 ± 2.66	29.08 ± 8.79	0.972
Butyric acid, %	11.87 ± 3.94	14.99 ± 5.72	11.68 ± 3.66	16.26 ± 3.31	0.252

^a–c^ Different superscripts within a row indicate a significant difference (*p* < 0.05). ^1^ The pen was considered as the experimental unit, with six replicate pens per treatment and five and four pigs per replicate pen in weeks 2 and 4, respectively. ^2^ CON—control diet was the basal ration; ABO—the basal plus 100 ppm of chlortetracycline hydrochloride, 120 ppm of colistin sulfate and 30 ppm of tiamulin hydrogen fumarate; ENZ—the basal plus 2000 IU/g of α-amylase, 5000 IU/g of β-glucanase, 3000 IU/g of protease and 4000 U/g xylanase; DFM—the basal plus 5 × 10^7^ cfu/g *Bacillus subtilis* and 2 × 10^6^ *Lactobacillus* spp.

**Table 8:** Effect of dietary treatment on fecal microbial population ^1^ (log10 cfu/g fresh feces) in weanling pigs at 2- and 4-weeks post-weaning ^1^.

	Week	CON ^2^	ABO	ENZ	DFM	*p*-Value
*Coliform*, cfu/g						
	0	7.24 ± 0.18	7.32 ± 0.20	7.48 ± 0.30	7.48 ± 0.20	0.185
	2	7.63 ± 0.19 ^a^	6.01 ± 0.23 ^c^	6.52 ± 0.12 ^b^	5.65 ± 0.21 ^d^	0.001
	4	7.33 ± 0.23 ^a^	5.19 ± 0.17 ^c^	5.71 ± 0.21 ^b^	5.66 ± 0.65 ^b^	0.001
	6	7.44 ± 0.16 ^a^	4.84 ± 0.06 ^c^	5.71 ± 0.20 ^b^	4.81 ± 0.13 ^c^	0.001
*E.coli*, cfu/g						
	0	7.43 ± 0.26	7.23 ± 0.35	7.25 ± 0.28	7.29 ± 0.30	0.667
	2	7.37 ± 0.18 ^a^	5.87 ± 0.13 ^c^	6.43 ± 0.40 ^b^	5.55 ± 0.23 ^c^	0.001
	4	6.92 ± 0.22 ^a^	4.93 ± 0.30 ^d^	5.71 ± 0.15 ^c^	6.04 ± 0.30 ^b^	0.001
	6	7.47 ± 0.20 ^a^	4.74 ± 0.22 ^c^	5.65 ± 0.13 ^b^	4.71 ± 0.12 ^c^	0.001

^a–d^ Different superscripts within a row indicate a significant difference (*p* < 0.05). ^1^ The pen was considered as the experimental unit, with six replicate pens per treatment and five and four pigs per replicate pen in weeks 2 and 4, respectively. ^2^ CON—control diet was the basal ration; ABO—the basal plus 100 ppm of chlortetracycline hydrochloride, 120 ppm of colistin sulfate and 30 ppm of tiamulin hydrogen fumarate; ENZ—the basal plus 2000 IU/g of α-amylase, 5000 IU/g of β-glucanase, 3000 IU/g of protease and 4000 U/g xylanase; DFM—the basal plus 5 × 10^7^ cfu/g *Bacillus subtilis* and 2 × 10^6^ *Lactobacillus* spp.

## Data Availability

The data that support the findings of this study are available on request from the corresponding author.
